# Our Sterilization Bill

**Published:** 1913-03

**Authors:** 


Our Sterilization Bill.—A simple and harmless method of cutting off the deluge of defectives, in part, at least, is found in vasotomy in the male, and fallopiotomy in the female, and it has become immensely popular, eight or ten States having adopted it in the State institutions. It has become a world movement, and is the greatest humanitarian reform of the century. We, in Texas, have for several years been endeavoring to secure such a law, but for various reasons have not been able to do so. Meantime, other States have gotten ahead of us. The bill has been published in the Texas Medical Journal every year, and our old readers are familiar with it. The one now before the Legislature, and which passed to engrossment in the House March 7th (inst.), is in no way different, except that it has been amended, and I add here the amendment, which I regard as superfluous and unnecessary:

"AMENDMENT (IN COMMITTEE ROOM).

"SEC. —. If any person or the friends or guardians of any person examined according to the provisions of this act shall object to the finding of the board, the said person or his or her friends or guardians shall have the right of appeal to the county court of the county in which the institution in which said person is legally confined is located. Said court to have the authority to summon and examine witnesses in chambers and to decide whether or not the finding of the board is in the interest of the State and society, which decision shall be final, and it shall be the duty of the superintendent of the institution in which the person herein granted the right of appeal is legally confined to notify the friends or guardians of said person of the time and place of said hearing by the county court, and, if no friends appear in behalf of the person demanding an appeal, it shall be the duty of the superintendent to notify the county judge of the county from which the person was sent of the name of the person and of the time, place and purpose of the hearing."

[That kills it; isn't worth a cent. It's a beginning (if it gets through), and can be bettered another time; maybe.—ED.]

